# Progress of Research on Conductive Hydrogels in Flexible Wearable Sensors

**DOI:** 10.3390/gels10020144

**Published:** 2024-02-14

**Authors:** Juan Cao, Bo Wu, Ping Yuan, Yeqi Liu, Cheng Hu

**Affiliations:** 1 School of Fashion and Design Art, Sichuan Normal University, Chengdu 610066, China; j.cao@sicnu.edu.cn; 2 School of Mechanical Engineering, Sichuan University, Chengdu 610065, China; scu_wubo@stu.scu.edu.cn (B.W.); 2022323020011@stu.scu.edu.cn (Y.L.); 3 School of Mechanical Engineering, Chengdu University, Chengdu 610106, China; yuanping@cdu.edu.cn; 4 National Engineering Research Center for Biomaterials, College of Biomedical Engineering, Sichuan University, Chengdu 610065, China

**Keywords:** conductive hydrogel, biomaterials, flexible sensors

## Abstract

Conductive hydrogels, characterized by their excellent conductivity and flexibility, have attracted widespread attention and research in the field of flexible wearable sensors. This paper reviews the application progress, related challenges, and future prospects of conductive hydrogels in flexible wearable sensors. Initially, the basic properties and classifications of conductive hydrogels are introduced. Subsequently, this paper discusses in detail the specific applications of conductive hydrogels in different sensor applications, such as motion detection, medical diagnostics, electronic skin, and human–computer interactions. Finally, the application prospects and challenges are summarized. Overall, the exceptional performance and multifunctionality of conductive hydrogels make them one of the most important materials for future wearable technologies. However, further research and innovation are needed to overcome the challenges faced and to realize the wider application of conductive hydrogels in flexible sensors.

## 1. Introduction

In recent years, wearable sensors have gained extensive attention and entered a period of booming development. Wearable sensors typically monitor body-related signals, such as body temperature, skin mechanics, breathing rate, pulse, blood pressure, body movement, electrophysiological signals, and biomacromolecule signals, through their layering onto human skin or embedding in wearable fabrics [1,2,3,4]. Wearable sensors have gradually evolved from single sensing devices that monitor human health to multifunctional, highly integrated, miniaturized, and diverse sensors [5,6] for continuous body signal monitoring, facilitating better information exchange with the environment. Compared to rigid sensors, sensors based on flexible substrates (such as fabrics [7], paper-based substrates [8], polymers [9], etc.) exhibit promising characteristics like stretchability, bendability, thinness, portability, and excellent electrical performance. Thus, a large number of wearable sensors based on flexible conductive materials have been developed. Traditional flexible wearable sensors are usually prepared by coating or filling flexible substrates (such as graphene [10], carbon nanotubes (CNTs) [11], silver nanowire (AgNW) [12], and Polyaniline (PANI) [13]) with conductive materials. Although their manufacturing methods are simple, the lack of sufficient interface interaction between the flexible substrate and the conductive filler not only limits the sensor’s strain response range but also causes a delamination of the conductive filler from the substrate, affecting the sensor’s signal conversion efficiency. Therefore, the development of flexible wearable sensing materials with good stretchability, high sensitivity, stability, and biocompatibility is particularly urgent.

**Figure 1:**
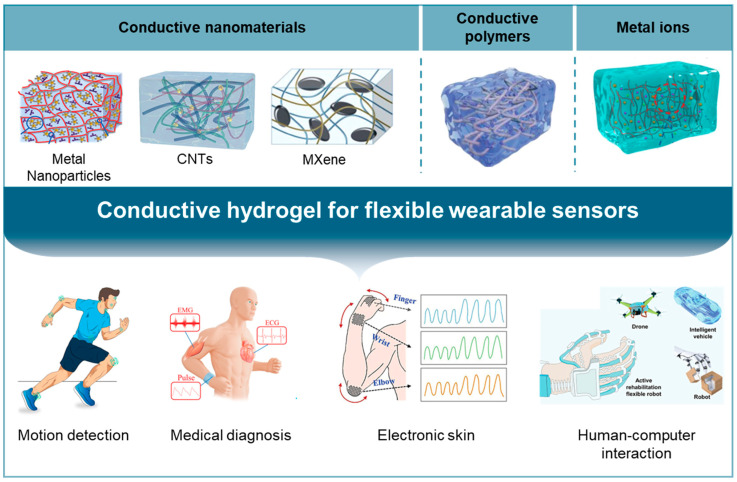
Different types of conductive hydrogels and their applications.

Currently, conductive hydrogels with a three-dimensional network structure, due to their high water content and excellent mechanical and extensible properties, are considered an ideal choice for flexible wearable sensors [14,15]. Moreover, conductive hydrogels also possess excellent biological properties like self-healing, self-adhesion, and antibacterial qualities [16,17,18], presenting unique advantages when tracking signals in close contact with biological tissues or organisms. Conductive hydrogels have become an active research area in the field of flexible electronics, demonstrating huge developmental potential in applications such as health monitoring [19], motion tracking [20], medical diagnostics [21], and human–machine interaction [22] (Figure 1), propelling our rapid entry into the era of “Internet of Everything”. Based on this, this paper discusses and systematically analyzes the characteristics, latest research, and application progress of flexible wearable sensors based on conductive hydrogels, and elaborates on their classification and research progress, including conductive nanocomposite hydrogels, conductive polymer composite hydrogels, and ionic conductive hydrogels. Subsequently, the application of conductive hydrogel-based flexible wearable sensors in motion detection, medical diagnostics, electronic skin, and HMIs is introduced. Finally, challenges and opportunities for future development are summarized and anticipated.

## 2. Types of Conductive Hydrogels

Hydrogels are polymers with a three-dimensional network structure, formed from natural or synthetic materials through different mechanisms such as physical entanglement, electrostatic interaction, and covalent chemical cross-linking. Their unique network structure endows hydrogels with high hydrophilicity, excellent biocompatibility, good viscoelasticity, and an ease of modification. A conductive hydrogel is prepared by introducing conductive components into the hydrogel. When it is used to make sensors, it can achieve a combination of flexibility and electronic transmission characteristics, thus achieving the combination of excellent mechanical properties, sensing characteristics, and biocompatibility. Based on different conductive fillers, the conductive hydrogels used in flexible wearable devices are classified into conductive nanocomposite hydrogels, conductive polymer hydrogels, and ionic conductive hydrogels, as shown in Table 1. Among them, the first two types of conductive hydrogels achieve electronic conduction by integrating conductive materials such as metal-based materials [23,24], carbon-based materials [25,26], and conductive polymers [27,28] into the hydrogel matrix to establish a network for electron transport. These hydrogels exhibit stable chemical properties and excellent electrical conductivity. The last type involves the introduction of ionic conductive materials (such as salt solutions [29,30], ionic liquids [31,32,33], etc.) into the hydrogel network. The three-dimensional network structure of the hydrogel provides pathways for ion migration, thereby facilitating the internal transport of free ions and ultimately achieving ionic conduction. These hydrogels possess good optical transparency, tunable mechanical properties, strain sensitivity, and stable electrical conductivity [34]. Furthermore, their excellent optical properties also facilitate their utilization within wearable sensors.

### 2.1. Conductive Nanocomposite Hydrogel

Incorporating conductive nano-fillers (such as metal nanomaterials [41], carbon nanomaterials [42], and MXene (Ti_3_C_2_T_X_, 2D transition metal carbides/carbon nitrides) [43], etc.) into hydrogel networks marks a significant breakthrough in the field of flexible sensors. Firstly, the detection sensitivity of conductive nano-hydrogels can be enhanced through the contact resistance effect and the tunneling effect [44]. Secondly, by adjusting the interactions between conductive fillers and the hydrogel matrix, the mechanical characteristics of hydrogels can be significantly improved [45]. Moreover, due to the distinctive attributes of conductive nanofillers, such as electromagnetic shielding, thermal conductivity, and photovoltaic effects, it is possible to design and create more flexible sensors endowed with multifaceted functionalities [46].

#### 2.1.1. Metal Nanomaterial-Based Composite Hydrogels

**Figure 2:**
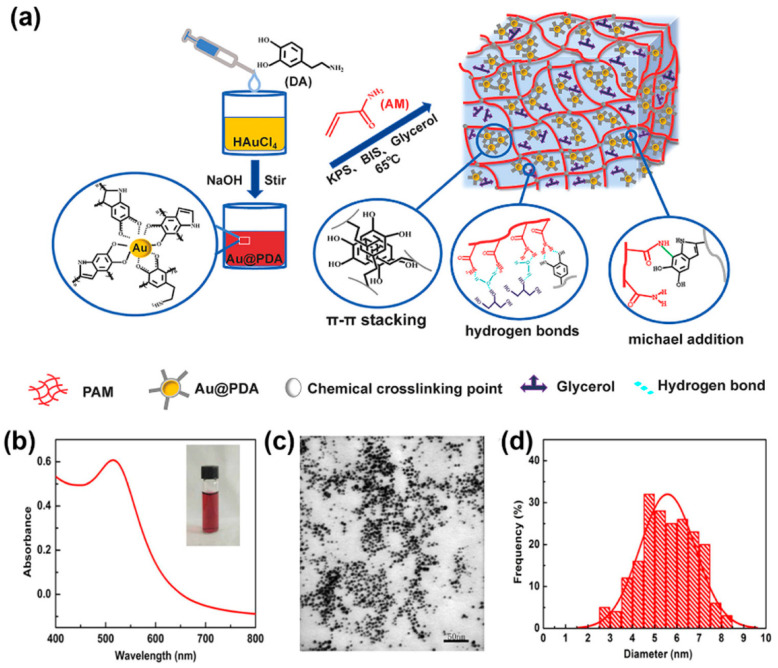
(**a**) The fabrication of PAM/Au@PDA hydrogel; (**b**,**c**) UV–visible absorption spectra and transmission electron microscopy images of Au@PDA nanoparticles; inset of (**b**) shows the dispersion of Au@PDA nanoparticles; (**d**) size distribution of AuNPs in the Au@PDA nanocomposite material. Copyright permission from Li et al. [24], *ACS Applied Materials & Interfaces*, 2019.

Metal nanomaterials, including metal nanoparticles [47], metal oxide nanoparticles [48], and metal nanowires [49], etc., possess excellent conductivity and high surface energy, making them ideal raw materials for preparing conductive composite hydrogel materials. Combining metal nanomaterials with hydrogels not only enlarges the specific surface area of the metal nanomaterials, enhancing the conductivity and mechanical properties of the hydrogels, but also confers to the metal materials a unique flexibility and ductility [50,51]. Traditional metal nanoparticles tend to aggregate and settle in the hydrogel matrix. To address this, Lu et al. [23] immobilized silver nanoparticles (Ag) on cellulose nanocrystals (CNC) modified with tannic acid (TA), utilizing the excellent dispersibility of CNC and its compatibility with hydrophilic polymers to achieve a good dispersion of Ag in a PVA matrix, creating a borax–silver/tannic acid@cellulose (PB-Ag/TA@CNCs) hydrogel which has good electrical and mechanical properties. Ag/TA@CNCs not only act as a conductive component and nano-reinforcement domain, significantly improving the gel’s conductivity (4.61 S m^−1^) and stretchability (>4000%), but also impart excellent antibacterial properties and repeatable self-adhesiveness, demonstrating promising application effects. Li et al. [24] introduced gold nanoparticles (AuNPs) functionalized with polydopamine (PDA) into polyacrylamide (Pam) hydrogels through the self-polymerization of dopamine (DA). Due to the excellent conductivity of AuNPs and the good dispersibility of PDA@Au NP, this hydrogel sensor demonstrated repeatable and stable electrical resistance signal responses to various magnitudes of human motion (Figure 2). Wang et al. [52] introduced silver nanoparticles (AgNPs) decorated with polydopamine (PDA)/cellulose nanofibers (CNF) into a polyacrylamide (PAm) network, creating a sensing material that features both tensile and compressive functions. Due to the strong conductivity of AgNPs, the hydrogel sensor exhibited high sensitivity to deformation, with a maximum gauge factor (GF) reaching 0.34. During 1000 cycles of stretching, the sensor showed stable and repeatable electrical signals. Additionally, the hydrogel with added AgNPs also possessed long-lasting antibacterial properties, beneficial for the extended use of a hydrogel sensor. However, it is important to note that the high cost associated with noble metal nanofillers leads to elevated research expenses. Additionally, certain metal nanomaterials are prone to corrosion in humid environments, which hinders their long-term utilization in extremely responsive environments.

While metallic nanomaterials can provide good conductivity to hydrogels, the inherent rigidity of metal materials might cause friction with the hydrogel matrix, leading to damage due to internal stress concentration. Recently, liquid metals (LM), such as eutectic gallium-based alloys, have been used as soft fillers in hydrogels to avoid damage to the hydrogel matrix, generating significant interest. For example, Yuan et al. [35] used a freeze–thaw method to prepare a composite flexible hydrogel sensor consisting of polyvinyl alcohol (PVA), tannic acid (TA), eutectic gallium–indium (EGaIn), and NaCl. The hydrogel exhibited good electrical conductivity, thermal conductivity, flexibility, and adhesiveness. Ultrasonic uniformly dispersed liquid metal EGaIn into the hydrogel network, greatly enhancing the hydrogel’s electrical conductivity (3.63 S m^−1^) and strain sensitivity (GF = 2.59). The sensor could stably monitor and distinguish various movements, such as joint bending, vocal cord vibration, and signals. Majidi et al. [53] reported a composite conductive hydrogel prepared by embedding a percolation network of Ag micro-flakes, eutectic gallium–indium (EGaIn), gallium–indium–tin (Galinstan), and other gallium-based liquid metal alloy droplets into polyvinyl alcohol–sodium borate (PVA–borax) gel. This hydrogel exhibits excellent self-healing and conductive properties. Due to its high liquid content, the composite material also demonstrates ideal mechanical properties, such as a low Young’s modulus (~20 kPa) and high stretchability (>400% strain).

#### 2.1.2. Carbon Nanomaterial-Based Composite Hydrogels

**Figure 3:**
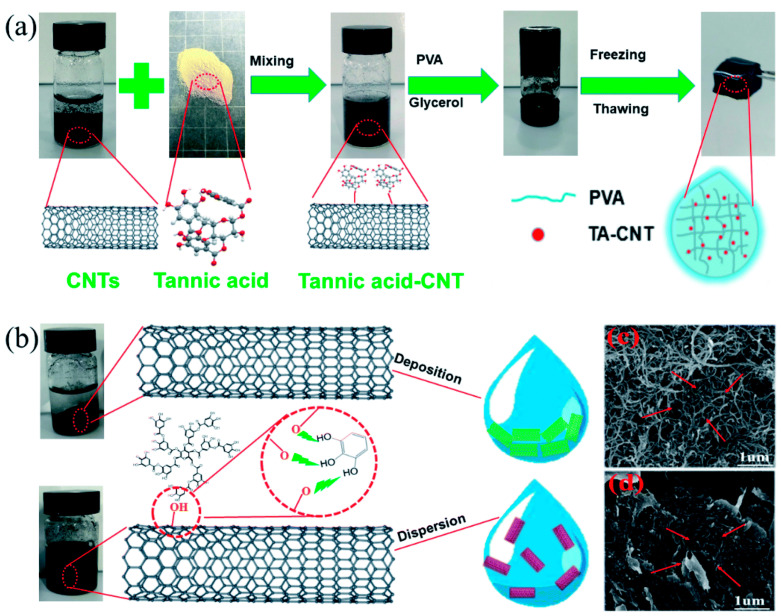
Preparation process of TCGP hydrogel and SEM images of CNTs and TA–CNT nanocomposites. (**a**) Illustration of the preparation of TCGP hydrogel. (**b**) TA-promoted dispersion of CNTs in solution. (**c**) SEM image of CNTs. (**d**) SEM image of TA–CNT nanocomposites. Copyright permission from Ni et al. [62], *Journal of Materials Chemistry A*, 2020.

Carbon nanomaterials such as graphene (GE) [54], carbon nanoparticles [55], carbon nanotubes (CNT) [56], graphene oxide (GO) [57], and carbon fibers (CF) [58] are ideal candidates for conductive hydrogel fillers due to their high specific surface area, excellent conductivity, and stability. The natural hydrophobicity of carbon-based materials like graphene and carbon nanotubes leads to their aggregation in aqueous media, hindering uniform and stable conductive systems within the hydrogel network. Based on previous studies, this issue can be effectively addressed using graphene oxide (GO) derived from graphite and reduced graphene oxide (rGO) [59], or by introducing hydrophilic compounds such as cellulose nanofibers (CNF) [60], and hydrophilic polymers [61]. For instance, Ni et al. [62] prepared a conductive hydrogel (TA-CNT-glycerol-PVA) by incorporating tannic acid–carbon nanotubes (TA-CNTs) into a polyvinyl alcohol (PVA) matrix containing a water–glycerol dispersion medium. This hydrogel exhibits excellent anti-freezing properties (−30 °C), long-term moisturization (10 d), and outstanding sensitivity (Figure 3). It can be used as a strain sensor for detecting various human movements and as an electrode for detecting electrophysiological signals, even in relatively harsh environments. Inspired by the multifunctionality of human skin, Fu et al. [25] incorporated carbon nanotubes (CNTs) into a chelate of calcium ions (Ca^2+^) with polyacrylic acid (PAA) and sodium alginate (SA), producing a conductive hydrogel with remarkable rheological properties, including stretchability, self-healing, and 3D printability. This hydrogel can be fabricated into integrated strain sensors with both piezoresistive and capacitive properties, responding sensitively to minute pressure changes of the human body, showing great potential in the field of flexible sensors. Xu et al. [63] developed a novel self-adhesive conductive organic hydrogel without catechol adhesion components by covalently crosslinking acrylamide, N-isopropylacrylamide, and reduced graphene oxide, followed by immersion in an ethylene glycol–water mixture. This hydrogel exhibits long-lasting moisturization (~30 days), extreme temperature resistance (−20–60 °C), stable conductivity, excellent stretchability (~1700%), high compressive stress (~5 MPa), and high strain sensitivity (gauge coefficient of 3.12), making it suitable for the long-term, continuous monitoring of human movements and electrocardiograms. Nevertheless, it is challenging to ensure the uniform dispersion of carbon-based nanomaterials within a system, and these limitations significantly restrict their potential applications in areas such as bioelectronics and electronic skin.

#### 2.1.3. MXene Composite Hydrogels

**Figure 4:**
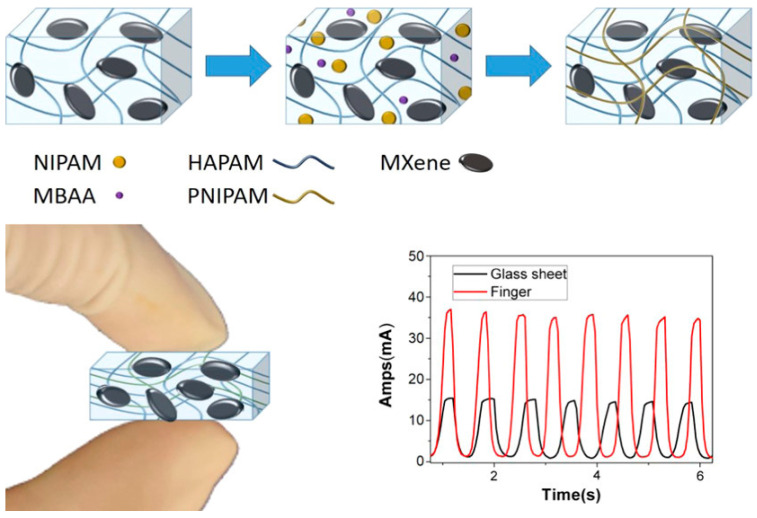
Schematic illustration of the network structure changes in the synthesis process of the NCDN hydrogel. Copyright permission from Ran et al. [70], *ACS Applied Materials & Interfaces*, 2019.

MXene, a two-dimensional nanomaterial based on transition metal carbides, nitrides, and carbonitrides, is obtained through the selective etching of MAX phases (like Ti_3_AlC_2_) with LiF/HCl and their subsequent ultrasonic dispersion [64,65]. With its unique layered structure, excellent metallic conductivity, abundant surface functional groups (-OH, -O, and -F) and hydrophilicity [66], MXene significantly enhances its interaction with the hydrogel network, forming stable conductive pathways and improving the hydrogel’s conductivity and sensing sensitivity [43,67]. In addition, the larger specific surface area and hydrogen bonding result in stronger interfacial interactions between MXene and the polymer matrix, which imparts higher mechanical strength to the hydrogel [68]. Therefore, MXene holds great potential in enhancing the electromechanical performance and sensitivity of hydrogels, showing promising applications in the field of flexible wearable sensors. To improve the performance of MXene-filled composite hydrogels, Liu et al. [69] proposed an oxidation method in an alkaline environment to customize the nanostructure of MXene, enhancing its dispersibility in the hydrogel and, consequently, the composite hydrogel’s conductivity, transparency, mechanical properties, and sensitivity. Ran et al. [70] used MXene nanosheets as conductive fillers and combined hydrophobically associated polyacrylamide (HAPAM) with temperature-sensitive poly(N-isopropyl acrylamide) (PNIPAM) to create a nanocomposite double-network hydrogel (NCDN) with dual sensing abilities for temperature and stress (Figure 4). The addition of MXene endowed the hydrogel with excellent mechanical properties and compressive strain conductivity. When combining compression and temperature sensing, the electrical signals of the hydrogel showed a significant numerical difference compared to pure compression, making it a candidate material for multifunctional sensors. Wang et al. [36] designed and synthesized a conductive hydrogel by in situ copolymerizing conductive surface-functionalized MXene (K-MXene)/Poly(3,4-ethylenedioxythiophene)/poly(styrenesulfonate) (PEDOT:PSS) ink with a thermos-responsive poly(N-isopropylacrylamide) (PNIPAM) hydrogel. In this system, the PNIPAM matrix provides mechanical flexibility, while the K-MXene/PEDOT:PSS ink acts as a conductive filler, mechanical enhancer, and photothermal agent. The hydrogel exhibited high electrical conductivity (11.76 S m^−1^), strain sensitivity (GF of 9.93), a wide operational strain range (≈560% strain), and high stability after more than 300 loading–unloading cycles at 100% strain. Chen et al. [26] introduced tannic acid-modified cellulose nanofibers (TA@CNF) and conductive MXene nanosheets into a covalently cross-linked polyacrylamide (PAAm) network permeated with a glycerol (Gly)–water binary solvent, creating a conductive nanocomposite organic hydrogel. This hydrogel exhibited excellent environmental stability, super-stretchability, self-adhesiveness, and self-repairing properties. Due to the abundant hydrogen bonding between water and Gly, the resultant organic hydrogel also exhibited significant low-temperature tolerance (down to −36 °C) and a long-term moisturizing ability (>7 d). These outstanding characteristics make it a promising candidate for wearable electronic sensors. Although MXene, as a two-dimensional nanofiller, exhibits excellent properties that can enhance the mechanical strength, electrical conductivity, and sensitivity of sensors, as well as provide unique features such as electromagnetic shielding, it is highly susceptible to oxidation in aqueous solutions, leading to the instability and even failure of the sensors. Therefore, the issue of oxidation failure of MXene in aqueous solutions is a critical challenge that needs to be addressed for its application in wearable sensors.

### 2.2. Conductive Polymer-Based Composite Hydrogels

**Figure 5:**
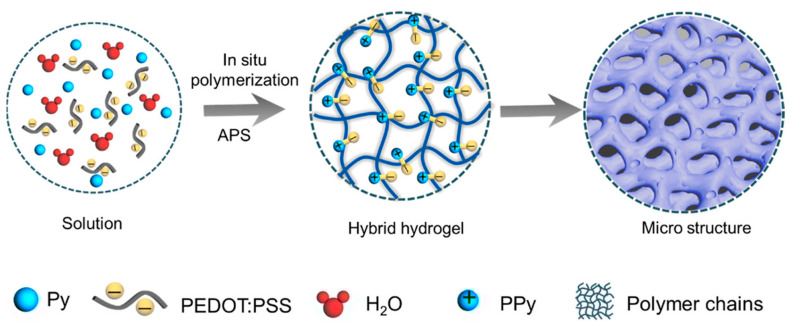
Fabrication process of PPy–PEDOT:PSS hybrid hydrogels. Copyright permission from Luo et al. [28], *ACS Applied Materials & Interfaces*, 2021.

Conductive polymers, primarily composed of carbon atoms and a conjugated π-electron system, transition from a semi-conductive or insulating state to a conductive state through p-type (hole) or n-type (electron) doping, endowing the polymers with a conductivity comparable to metals and other beneficial properties derived from polymer characteristics (e.g., mechanical strength, stability, and biocompatibility) [71,72,73]. Common conductive polymers include Polyaniline (PANI) [74,75], Polypyrrole (PPy) [71,76], Polythiophene, and Poly(3,4-ethylenedioxythiophene)/polystyrenesulfonate (PEDOT:PSS) [77,78,79]. Due to the controllability achieved through the commonly employed doping methods, conductive polymers exhibit tunable electrical properties. Taking advantage of their adjustable conductivity and straightforward fabrication processes, conductive polymers are often introduced as conductive fillers into hydrogel networks for applications in wearable and implantable electronic devices. Ren et al. [27] embedded polypyrrole particles in a hydrogel composed of iron ions (Fe^3+^), cross-linked acrylic acid, and chitosan polymer, developing a conductive hydrogel with high electrical conductivity (2.61 S m^–1^) and good mechanical properties (tensile strength of 628%, stress of 0.33 MPa, elastic modulus of 0.146 MPa, and toughness of 1.14 MJ m^–3^). Moreover, this hydrogel demonstrated a 93% self-healing efficiency within 9 h in air, without any external stimuli, showing promise for a wide range of new soft material applications. Luo et al. [28] mixed a pyrrole monomer with a PEDOT:PSS dispersion and then performed in situ chemical oxidative polymerization to form PPy. The electrostatic interaction between negatively charged PSS and positively charged conjugated PPy facilitated the formation of a PPy-PEDOT:PSS hybrid hydrogel (Figure 5). This hydrogel exhibited an electrical conductivity of 867 S m^−1^ and excellent biocompatibility. Its multi-porous structure was beneficial for 3D cell culturing within the hydrogel, and its outstanding in situ biomolecule detection and real-time cell proliferation monitoring properties indicate its great potential in high-sensitivity electrochemical biosensors. Chen et al. [38] developed a flexible hydrogel sensor based on a dual synergistic network composed of poly(acrylic acid) (PAA) and conductive PANI chemically doped with phytic acid (PA). Due to the physical entanglement, hydrogen bonding, and ionic interactions between the conductive PANI network and PAA, the resulting hydrogel sensor exhibited high tensile strength (0.3 MPa), excellent elongation at break (1160%), and good fatigue resistance. Furthermore, with the addition of conductive PANI, the hydrogel sensor exhibited controllable electrical conductivity (0.03~5.12 S m^−1^), good sensing sensitivity (GF = 1.05), and a wide strain sensing range (0~1130%).

### 2.3. Ionic Conductive Hydrogels

**Figure 6:**
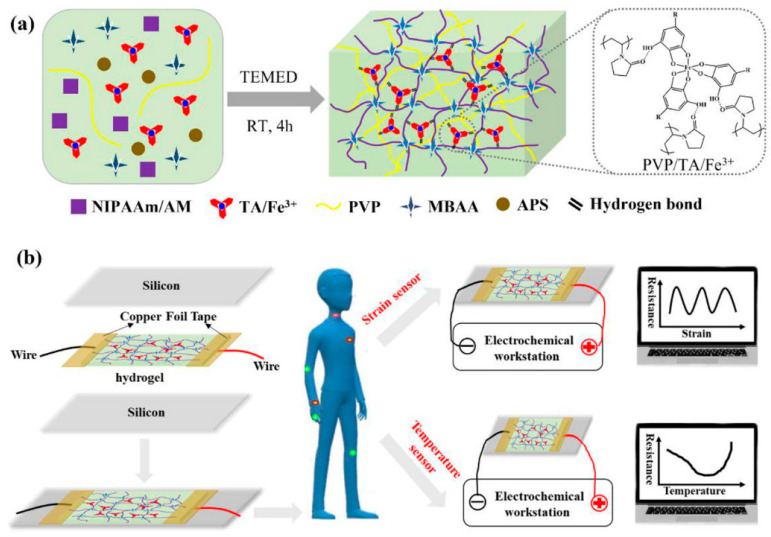
(**a**) Design of the temperature-responsive P(NIPAAm-AM)/PVP/TA/Fe^3+^ conductive hydrogel and (**b**) its potential to be used as a wearable strain sensor and temperature sensor to monitor human motions and detect abnormal hyperthermia of the human body. Copyright permission from Ma et al. [32], *ACS Applied Materials & Interfaces*, 2022.

Hydrogels, with their three-dimensional network structures, allow carriers to freely migrate within them. The introduction of ions such as Li^+^, Na^+^, Fe^3+^, and Al^3+^ into hydrogels [80,81,82,83] can provide hydrogels with stable electrical conductivity, which is a unique advantage. Unlike adding conductive polymers or nanomaterials, ionic conductive hydrogels are typically transparent, which is beneficial for visualizing the interiors of electronic devices [49,84]. Xu et al. [31] dissolved cellulose directly in an ion solution containing Zn^2+^ and Al^3+^ and then cross-linked it with an acrylic acid (AA) and acrylamide (AAm) copolymer to form multiple hydrogen bonds, creating an Ion-C-P (AA-co-AAm) conductive hydrogel. This hydrogel exhibited excellent swelling resistance (88.03%) and compression properties (24.11 MPa), and, due to the abundance of Zn^2+^ and Al^3+^, it also possessed remarkable conductivity (48.39 mS cm^−1^) and freezing resistance, offering new perspectives for expanding hydrogel applications in flexible electronics. Ma et al. [32] developed a novel dual-network ionic conductive hydrogel by introducing polyvinylpyrrolidone (PVP)/tannic acid (TA)/Fe^3+^ (Figure 6). In this hydrogel, PVP/TA/Fe^3+^ formed a crosslinked network through hydrogen bonding and metal coordination, and it interlocked with the P(NIPAAm-co-AM) network, enhancing the flexibility, strength, and conductivity of the hydrogel. This hydrogel has good stretchability (720%), a rapid response time (265 ms), excellent conductivity (0.79 S m^−1^), temperature sensitivity, transparency, and viscosity. These characteristics highlight its potential as a wearable dual strain and temperature sensor. Wang et al. [29] added trehalose and LiCl to a P(AM-co-AA) polymer network, creating a multifunctional ionic conductive hydrogel. The covalent hydrogen bonding interactions and strong hydration of LiCl significantly improved the mechanical properties of the hydrogel, achieving a maximum elongation at a break of 4529% under tensile strain. LiCl endowed the hydrogel with excellent ionic conductivity. The P(AM-co-AA)/trehalose/LiCl ionic hydrogel exhibited good self-adhesiveness to various substrates, along with outstanding anti-freezing and moisture retention properties, maintaining high stretchability and conductivity at −20 °C. The assembled hydrogel strain sensor exhibited excellent sensitivity (GF = 3.59% in the strain range of 2200–4200%). This strain sensor could sensitively and accurately detect joint bending, facial expressions, and swallowing behaviors, showing broad application prospects. Although ionic conductive hydrogels, which incorporate free moving ions, have conclusive advantages in their electrochemical performance, mechanical properties, and even unique high transparency, providing a crucial foundation for their development in flexible wearable sensors, current ionic conductive hydrogels still suffer from the drawback of ion leakage. This places them at a disadvantage in terms of electrochemical stability. Therefore, the development of ion-conductive hydrogels with an excellent ion retention capability will play a critical role in shaping their future advancements.

## 3. Applications in Flexible Wearable Sensors

Due to their excellent biocompatibility and unique network structure, hydrogels are considered the ideal choice for fabricating flexible sensors. Flexible sensors based on conductive hydrogels not only require outstanding conductivity and sensitivity but also high mechanical strength to ensure that they do not easily break under substantial mechanical loads [85,86]. Their self-repairing ability [87,88,89] is a crucial characteristic which ensures that sensors can still stably transmit electrical signals after experiencing mechanical damage, thereby extending the sensor’s lifespan and reducing electronic waste. Additionally, hydrogel sensors should possess adhesiveness [90,91], and establish a stable and reliable contact interface with human tissues to capture small physiological signals without the use of adhesive tape. More importantly, hydrogel sensors using water as a dispersion medium can experience water evaporation and low-temperature freezing hardening, severely affecting the sensor’s long-term stability. Therefore, anti-freezing and moisture retention properties [92] are also essential characteristics to consider when developing flexible conductive hydrogel-based sensors. In recent years, a series of conductive hydrogels with unique advantages such as good stretchability, stable conductivity, biocompatibility, mechanical robustness, anti-freezing moisture retention, and antibacterial properties have been prepared using different synthesis methods and materials. These have been extensively researched and applied in areas such as motion detection, medical diagnostics, electronic skin, and human–machine interactions [93], as shown in Table 2.

### 3.1. Motion Detection

**Figure 7:**
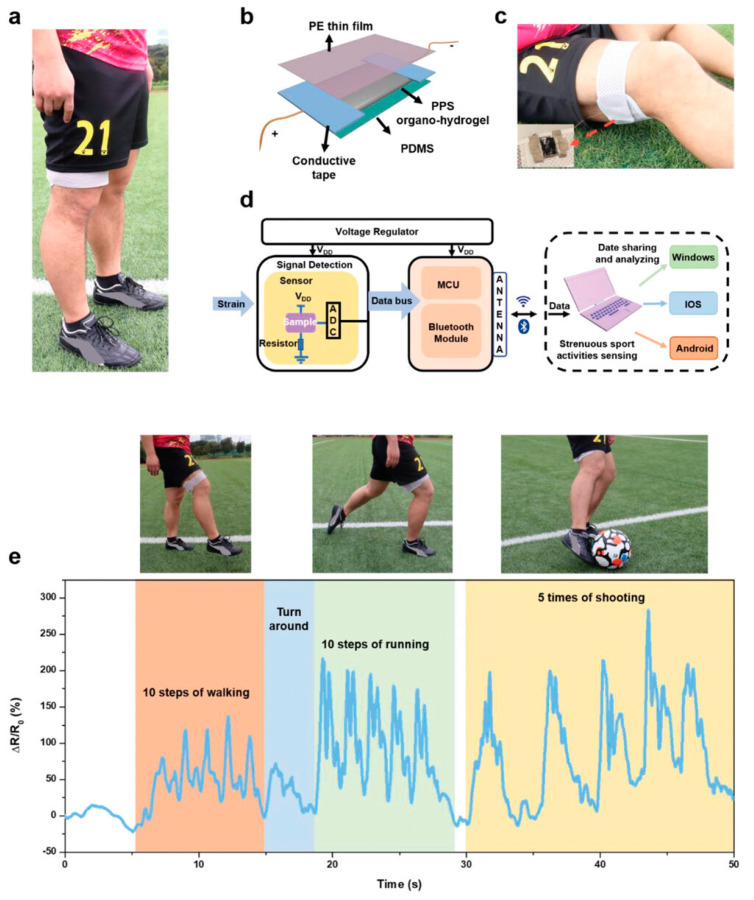
Monitoring signals for intense movements. (**a**) Application in soccer activities. (**b**) Sensor device structure. (**c**) Electronic wristband for detecting thigh signals. (**d**) Flowchart for monitoring soccer activities. The PPS organic hydrogel senses and collects motion data, transmitting digital signals to external devices controlled by a Bluetooth chip. (**e**) Recording of movement signals such as walking, running, and shooting during soccer training. Copyright permission from Zhai et al. [109], *Advanced Materials*, 2023.

In recent years, conductive hydrogels have been endowed with rapid responses and high strain/pressure sensitivity, enabling the real-time monitoring of various human movements [107]. Han et al. [108] developed a novel conductive organic hydrogel for strain sensing, mainly composed of tannic acid (TA)-coated cellulose nanocrystals, graphene, borax, and polyacrylamide (PAm), and partially replaced the water in the network with ethylene glycol (EG) through a simple solvent substitution strategy. This hydrogel sensor exhibits an excellent surface adhesion performance (220 KPa on pigskin surface) and mechanical properties (elongation at break of 3734%, breaking strength of 0.47 MPa). It also shows a sensitive and stable sensing performance, excellent reliability, and a wide sensing range (5–1500%), accurately monitoring human joint movements, wrist pulse, micro-expressions, and vocal signals. These capabilities demonstrate its vast potential in intelligent motion recognition for posture correction and rehabilitation. Gao et al. [20] used ionic liquids as raw materials to construct a multifunctional polyvinyl alcohol (PVA)/carboxymethyl cellulose (CMC)/poly(acrylamide-co-1-vinyl-3-butylimidazolium bromide) (P(AAm-co-VBIMBr)) (PCPAV) ionic conductive hydrogel. The prepared ionic conductive hydrogels have multiple crosslinked network structures, multiple hydrogen bonding interactions, and electrostatic interactions, demonstrating excellent tensile properties (810.6%), significant tensile stress (360.6 kPa), good toughness, and fatigue resistance. The introduction of VBIMBr endowed the PCPAV hydrogel with high ionic conductivity (15.2 mS cm^−1^), exceptional transparency (~92%), and frost resistance (−45.5 °C). The flexible sensors assembled using this hydrogel exhibited high strain sensitivity (GF = 3.75), fast responses, long-term stability, and durability, capable of monitoring various large-scale human joint movements and detecting minute muscle movements. Zhai et al. [109] prepared a robust conductive multi-structured PEDOT:PSS/PVA organic hydrogel (PPS organic hydrogel) through a simple strategy combining self-assembly and stretching training. The PPS organic hydrogel had a PVA/PEDOT:PSS layered structure and aligned PVA/PEDOT:PSS nanofibers, PVA, and PEDOT:PSS nanocrystalline domains, and a semi-interpenetrating network structure, displaying excellent mechanical properties (strength: 54.8 MPa, toughness: 153.97 MJ m^−3^). Moreover, due to its orderly multi-layered structure and DMSO organic liquid phase composition, the PPS organic hydrogel also exhibited strong sensing properties (GF: 983). The PPS organic hydrogel was applied to an electronic wristband to monitor movement signals in soccer activities (Figure 7), including walking, running, shooting, and dribbling. The results showed the outstanding mechanical and sensing performance of the PPS organic hydrogel and its tremendous potential in detecting signals from intense movements. The compatibility issues between the conductive hydrogel matrices and their swelling behavior in humid environments significantly affect the mechanical and electrical performance of conductive hydrogels, limiting their applications in wearable electronic devices. Therefore, Li et al. [37] developed a polyacrylamide-alginate-polypyrrole (PAM-ALG-PPy) CPH with high strength and rigidity and excellent anti-swelling properties by combining the supramolecular interactions between hard conductive polymers (PPy) and soft materials (PAM-ALG), including hydrogen, coordinate bonds, and cation–π interactions. Benefiting from the effective interactions between the polymer networks, the resulting supramolecular hydrogel exhibited uniform structural integrity, significant tensile strength (1.63 MPa), outstanding elongation at break (453%), and remarkable toughness (5.5 MJ m^−3^). As a strain sensor, the hydrogel has high conductivity (2.16 S m^−1^), a wide linear strain detection range (0–400%), and excellent sensitivity (gauge factor = 4.1), capable of the real-time monitoring of human movements under both large and small strains, showing significant broad sensing capabilities.

### 3.2. Medical Diagnostics

With an increasing focus on personal health, using hydrogel sensors for the real-time monitoring of physiological activities (such as pulse, respiration, and heartbeat) has become a hot topic, and the data collected can be used for the early diagnosis of diseases. Wu et al. [110] developed a polyaspartic acid-modified dopamine/ethyl ionic liquid hydrogel (PDEH). By further embedding a silver liquid metal (SLM) conductive layer to create PDEH-SLM patches, they could capture electromyographic signals for diagnosing peripheral neuropathies. Through a one-step electrical field treatment, the hydrogel achieved rapid and extensive adhesion regulation and greatly enhanced mechanical properties. Moreover, the hydrogel patch assembled with the silver liquid metal (SLM) layer exhibited excellent charge injection and low contact impedance, capable of capturing high-fidelity electromyographic signals. This work further validates the feasibility of conductive hydrogel devices for the accurate diagnosis of sensory, motor, and mixed peripheral neuropathies.

**Figure 8:**
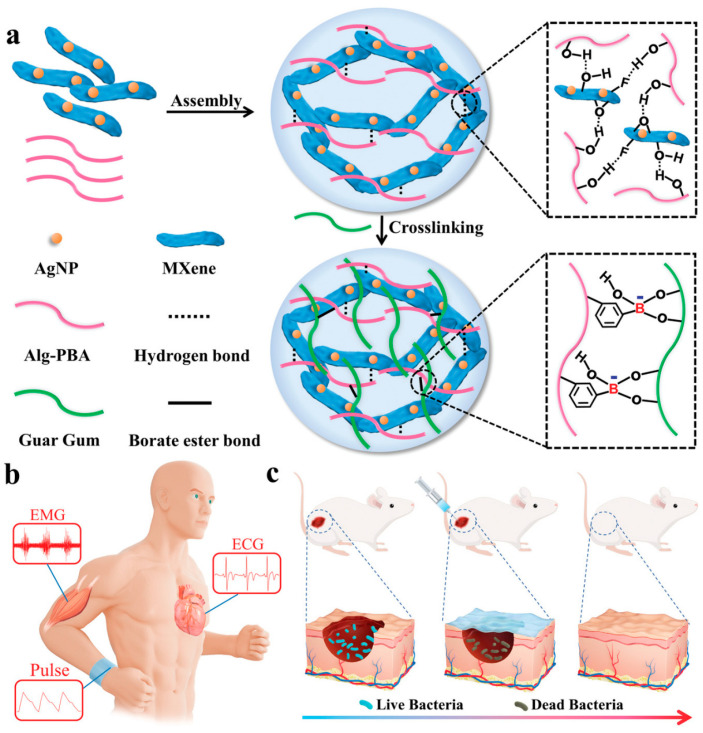
(**a**) Schematic diagram of the fabrication of the healable, injectable, and antibacterial MXene hydrogel and its further applications in (**b**) human health monitoring and (**c**) wound treatment. Copyright permission from Wan et al. [102], *Advanced Functional Materials*, 2022.

Various flexible epidermal sensors based on conductive hydrogels have made great progress in human health monitoring. However, the development of integrated health devices that combine reliable, sensitive diagnostic properties and timely treatment remains a great challenge in the wearable sensor field. Wan et al. [102] developed a healable, injectable, and antibacterial MXene-based hydrogel by incorporating MXene nanosheets modified with antibacterial silver nanoparticles (AgNPs/MXene) into a polymer network of guar gum (GG) and phenylboronic acid-grafted sodium alginate (Alg-PBA). This MXene hydrogel (AgNPs/MXene/GG/Alg-PBA) is suitable for wearable human–machine interactions and high-performance human health monitoring applications (Figure 8). The introduction of antibacterial AgNPs/MXene nanosheets into the hydrogel resulted in enhanced mechanical strength, improved conductivity, and strong antibacterial properties. The MXene hydrogel was fabricated into a multifunctional skin sensor, capable of sensitively monitoring human activities and detecting minute electrophysiological signals such as electrocardiograms (ECG) and electromyograms (EMG), thereby providing crucial clinical information for rehabilitation training as well as cardiovascular and muscular diseases. Additionally, its excellent self-healing ability, powerful injectability, and reliable antibacterial performance allow the MXene hydrogel to be directly injected into wound sites for further antibacterial action and wound healing, effectively accelerating wound recovery and showing great potential in wearable electronics, health diagnostics, and smart medicine. Similarly, Yang et al. [21] designed a multifunctional PAAm/PEG/hydrolyzed keratin/MXene conductive hydrogel (PPHM hydrogel) as a high-performance therapeutic integrated epidermal sensor. This sensor exhibits high-sensitivity sensing properties (thickness factor at high strain = 4.82), strong mechanical tensile properties (up to 600% maximum elongation at break), a rapid self-healing ability, stable self-adhesiveness, biocompatibility, −20 °C frost resistance, and an adjustable photothermal conversion capability. The PPHM hydrogel can sensitively monitor human movements and detect minute electrophysiological signals to diagnose related activities and diseases. It can also serve as an effective wound dressing to accelerate the healing process, providing valuable insights for developing integrated diagnostic and therapeutic smart wearable devices. Hou et al. [111] reported a new synthesis strategy for an ionic conductive hydrogel enhanced with core–shell structured curcumin nanoparticle composites for simultaneous cardiac electrophysiological signal monitoring and myocardial infarction repair. Zhang et al. [112] developed a conductive hydrogel with a pNIPAm and poly(Cu-arylacetylide) interpenetrating polymer network, exhibiting exceptional anti-swelling properties, good electronic conductivity, good adhesion, and excellent antibacterial performance. This hydrogel is capable of recording electrocardiograms (ECG), electromyograms, implantable epicardial ECGs, and transmitting neural signals. Moreover, the Cu(I) in the polymer chains can be replaced by other metal ions such as Au(I), creating more high-performance new materials. This research not only opens up new areas of study for hydrogels but also proposes the concept of designing implantable electrodes for the recording of bioelectronics.

### 3.3. Electronic Skin

**Figure 9:**
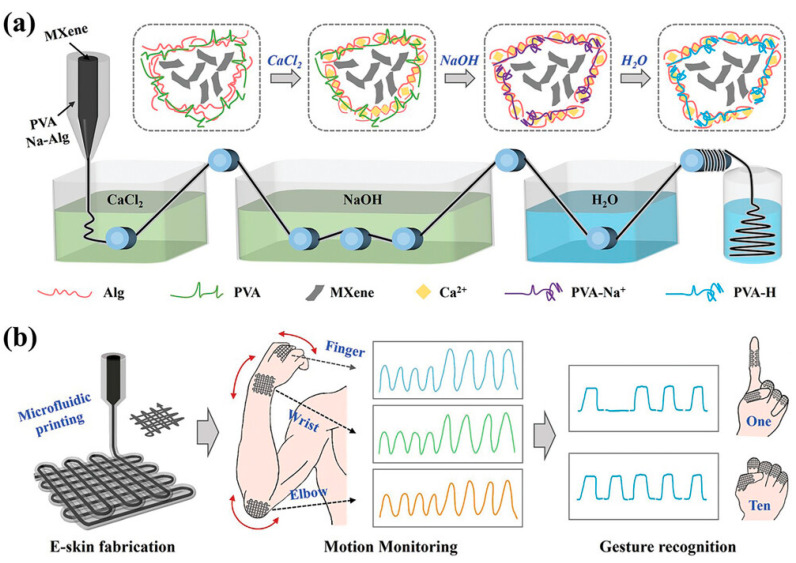
A schematic illustration for the fabrication and application of conductive hydrogel microfibers. (**a**) The process and reaction principle of preparing microfibers. (**b**) Microfluidic printed e-skin for motion monitoring and gesture recognition. Copyright permission from Wang et al. [115], *Small*, 2023.

Electronic skin (e-skin) is a type of flexible electronic device that can adhere to the surfaces of various objects, mimicking the tactile sensing function of human skin. It converts physical, chemical, and physiological signals such as strain, pressure, temperature, humidity, and bodily fluids into electrical signals. E-skin has advantages like stretchability, lightweight, and good biocompatibility [113]. The pliability and high sensitivity of conductive hydrogels make them prime candidates for e-skin applications [93]. Inspired by the structure of human skin, Guan et al. [114] constructed an e-skin with a sandwich structure for health monitoring purposes. The sensor, comprising a first network of polyacrylamide-co-acrylic acid and a second network of PVA, achieved high strain-sensitive conductivity and adhesiveness. The hydrogel, obtained through traditional one-pot methods, exhibited good mechanical tunability due to its glycerol content and DN structure. The mixture of graphene oxide (GO) and carbon nanotubes (CNTs) was coated onto the hydrogel by spraying. The crack mechanism of the CNTs/GO in the cracking process endowed the sensor with excellent sensing performance, such as ultra-high sensitivity (GF = 20) and remarkable durability and stability over a wide strain range (0–300%). Additionally, the adhesive layer, serving as an interface layer between the sensing element and human skin, contains multiple functional groups that can interact with different materials. The adhesive layer enables the sensor to adhere to the skin, detecting minute strains such as throat vibrations and heart rates, providing a viable solution for improving wearable electronic devices for healthcare monitoring. Wang et al. [115] proposed a method for fabricating conductive hydrogel microfibers using a one-step microfluidic-based method, aimed at constructing super-stretchable electronic skin. The microfibers consist of a conductive MXene core and a hydrogel shell. The core is solidified through the covalent cross-linking of sodium alginate and calcium chloride, while the shell is mechanically reinforced by a complexation reaction between polyvinyl alcohol and sodium hydroxide (Figure 9). By adjusting the flow rates and concentrations of the core and shell liquids, the conductivity of the microfibers can be customized. Due to the significant advantages of the superfine fiber in terms of mechanical and electrical properties, the resulting electronic skin exhibits impressive stretchability and sensitivity, which also shows attractive application value in motion monitoring and gesture recognition. Liu et al. [116] employed a one-pot method, using natural collagen fibers as a 3D network framework, and introduced a mixture of betaine, AgNPs, sodium chloride (NaCl), and a glycerol–water binary solvent to design a new multifunctional natural-skin-based organic hydrogel electronic skin (NSD-Gel e-skin). The NSD-Gel e-skin has excellent transparency, tensile strength (7.33 MPa), puncture resistance, moisturizing properties, and antibacterial properties. Additionally, the NSD-Gel e-skin exhibits excellent cold/heat resistance and stimulus-responsive properties, effectively sensing changes in environmental temperature or humidity, as well as monitoring human physiological/motion signals. In vitro and in vivo experiments have shown that the NSD-Gel e-skin has ideal biocompatibility and can protect tissues even in harsh environments (−196 °C to 100 °C).

### 3.4. Human–Machine Interactions

**Figure 10:**
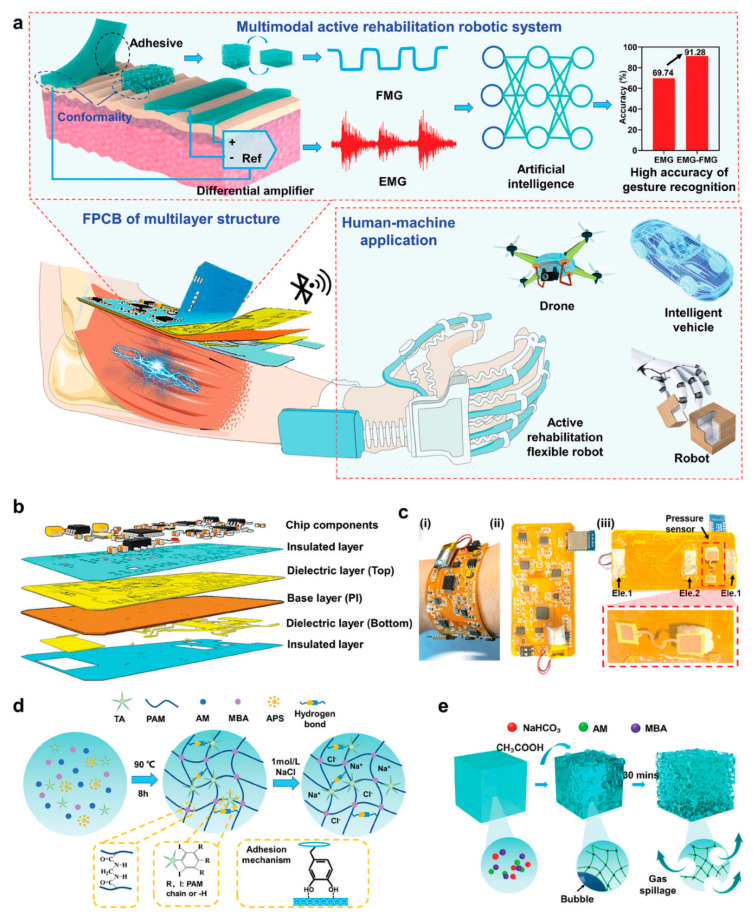
Design of a wearable human–machine interface. (**a**) Schematic of the wearable human–machine interface based on hydrogel electromyography and pressure sensors, and the corresponding AI-assisted intelligent active rehabilitation robotic system, showing wide application prospects in human–machine applications. (**b**) Schematic of the self-designed multi-layer structure of the FPCB. (**c**) Photographs showing the FPCB in contact with and conforming to human skin curvature (i); the front view of the HMI (ii); and the distribution of sensors on the HMI (iii). (**d**) Schematic of the fabrication strategies for NaCl-TA-PAM hydrogel and (**e**) Foam-PAM hydrogel. Copyright permission from Wu et al. [120], *Advanced Materials*, 2023.

Human–machine interfaces (HMIs) are vital mediums for the interactions between humans and computers, virtual reality systems, intelligent devices, etc. With the advent of the intelligent era and the increasing requirement for more intuitive, natural, and enriching interaction experiences, research and innovation in HMIs have become crucial. The main challenge of HCIs comes from the materials, and hydrogels’ unique similarity to biological tissues and their multifunctionality, softness, and malleability in terms of customizing properties make them continually evolving and potent in human–machine interface research and applications [34,117]. Tan et al. [118] developed and prepared a series of conductive hydrogels (C_x_P_y_) based on chitosan, water-soluble polypyrrole, and cucurbituril [7]. These C_x_P_y_ hydrogels exhibit good mechanical strength (215.48 kPa, strain at break 2149.17%), excellent adhesion strength (~51.54 kPa), remarkable conductivity (0.534 S m^−1^), and biocompatibility (cell viability of NIH3T3 is 98.25%). As strain sensors, C10P5 hydrogels showed excellent stability over 1000 cycles, suitable for epidermal sensors monitoring body movement and physiological signals. These conductive hydrogels demonstrate broad potential in intelligent health monitoring and human–machine interfaces. Zhao et al. [119] employed a free radical polymerization strategy to prepare a MXene/polyacrylic acid (PAA) hydrogel, constructing a flexible strain sensor that features high sensitivity (gauge factor ≈ 4.94), a wide detection range (0–1081%), and photo-thermal conversion properties. This sensor can rapidly detect human motion, accurately capture small-strain physiological activities such as frowning, smiling, and throat movement, and remotely control manipulators to perform simple open–close actions. It can also sense minute deformations, achieving continuous hovering and following motions. As a proof of concept, this work has inspired the further development and application of multifunctional MXene/PAA hydrogels in the field of human–machine interactions. The HMI is a bridge for human–computer communication. To achieve more flexible and comfortable interactions, Wu et al. [120] developed a wearable integrated human–machine interface, consisting of multimodal sensing modules and a flexible printed circuit board (FPCB). The multimodal sensing module integrates epidermal electrodes and pressure sensors, made, respectively, of TA-modified (NaCl-TA-PAM) and macroporous structured PAM hydrogels (Foam-PAM) (Figure 10). Due to the individual modulation and coupling of the components and the structure of the epidermal electrode hydrogel, it exhibits excellent adhesiveness, conductivity, and biocompatibility, enabling the stable and reliable monitoring of epidermal electrophysiological signals. The pressure sensor, prepared by a simple gas foaming method, shows high sensitivity to weak pressure (0.95 kPa^−1^ in the range of 16 to 448 Pa) and can be used to monitor minute FMG signals, significantly enhancing the efficiency of human–machine interactions and offering broad application prospects.

## 4. Conclusions and Future Perspectives

In recent years, wearable sensors have seen rapid development. As ideal materials for the fabrication of flexible wearable sensors, conductive hydrogels have attracted increasing attention, achieving significant progress in their research and applications. Conductive hydrogels based on different conductive materials (conductive nanocomposite hydrogels, conductive polymer hydrogels, and ionic conductive hydrogels) possess excellent biocompatibility, high conductivity, mechanically tunable properties, and customizable self-healing, self-adhesion, frost resistance, and moisturizing properties, and antibacterial advantages. These qualities have sparked great interest in their applications in motion detection, medical diagnostics, electronic skin, and human–machine interactions, offering vast possibilities for their future development in human–machine interfaces and wearable devices.

Despite the widespread application of conductive hydrogels in flexible wearable sensors, their future practical applications still face several challenges: (1) The long-term stability of conductive hydrogels needs further improvement to cope with the effects of prolonged use and environmental changes on the material properties. Although there are some strategies to enhance the mechanical properties of the conductive hydrogel to improve the mechanical stability of the conductive hydrogel, the evaporation of water in the hydrogel will still lead to performance degradation as the use time increases. Therefore, maintaining the long-term stability of conductive hydrogels remains a key challenge. (2) In the development of conductive hydrogels for flexible wearable sensors, sensitivity and selectivity are two very important factors. They determine whether the sensor can accurately detect and respond to specific biological or physical signals. As the requirements for the multifunctionality and integration of flexible wearable devices increase, enhancing the sensitivity of conductive hydrogels and optimizing their selectivity for specific physiological signals remains a challenging and ongoing task. (3) Finally, although conductive hydrogels typically exhibit good biocompatibility, the biosafety, degradability, and recyclability of flexible sensors based on the conductive hydrogels in practical use still require in-depth exploration to ensure their effective and safe application. This notwithstanding, the future development prospects of conductive hydrogels in flexible wearable sensors are highly promising. First of all, to improve the stability and water resistance of conductive hydrogels it is necessary to actively explore new synthesis methods. For example, the introduction of crosslinking agents or the modification of the material structure can enhance the water resistance of conductive hydrogels, reducing the risk of performance degradation in humid environments. Additionally, it is also possible to consider introducing multifunctional components such as optical, thermal, or bioactive substances into conductive hydrogels to achieve their broader application in flexible wearable sensors. Furthermore, with the increasing emphasis on sustainability and environmental concerns, researchers should explore the development of renewable materials or biodegradable alternatives to conductive hydrogels. These materials can reduce our dependence on finite resources and minimize our environmental impact.

In summary, the research on and application of conductive hydrogels in flexible wearable sensors is progressing rapidly, offering new possibilities for realizing comfortable, highly sensitive, and multifunctional sensors. The ongoing development and innovation in this field are expected to generate more applications and opportunities for hydrogels in human health monitoring, intelligent medicine, and human–machine interactions.

## Figures and Tables

**Table 1:** Properties of conductive hydrogels based on different conductive materials.

Type	Conductive Materials	Hydrogel Networks	Optical Property	Conductivity	References
Conductive nanomaterials	Ag nanoparticles	PVA/CNC	Black	4.61 S m^−1^	[23]
Conductive nanomaterials	Au nanoparticles	PAm	Black	-	[24]
Liquid metal	EGaIn	PVA/TA	Black	3.63 S m^−1^	[35]
Conductive nanomaterials	CNTs	PAA/SA	Black	22.5 S cm^−1^	[25]
Conductive nanomaterials	MXene/PEDOT:PSS	PNIPAM	Black	11.76 S m^−1^	[36]
Conductive nanomaterials	MXene	PAAm	Black	1.9 mS cm^−1^	[26]
Conductive polymers	PPy	AC/chitosan	Black	2.61 S m^−1^	[27]
Conductive polymers	PPy/PEDOT:PSS	PPy/PSS	Black	867 S m^–1^	[28]
Conductive polymers	PPy	PAM-ALG	Black	2.16 S m^−1^	[37]
Conductive polymers	PANI	PAA/PA	Black	0.03~5.12 S m^−1^	[38]
Conductive polymers	PDA/PPy	PAm	Transparent	12 S m^−1^	[39]
Conductive polymers	PANI	PVA	Black	1.7 mS cm^−1^	[40]
Metal ion	Zn^2+^/Al^3+^	AA/AAm	Transparent	48.39 mS cm^−1^	[31]
Metal ion	Fe^3+^	PVP/TA	Transparent	0.79 S m^−1^	[32]
Metal ion	LiCl	P(AM-co-AA/SA	Transparent	-	[29]
Metal ion	Fe^3+^	CS-P(AM-*co*-AA)	Brown	0.31 S m^−1^	[33]
Metal ion	LiCl	PVA/PEI	Transparent	11.76 S cm^−1^	[30]

**Table 2:** Different applications and properties of conductive hydrogels in flexible wearable sensors.

Applications	Synthesis Processes	Working Components	Characteristics	References
Motion detection	One-step thermal initiation	Cardanol/acrylic acid	Superelastic/anti-freezing/antidrying	[94]
Motion detection	Solvent-replacement strategy	Ethylene glycol (Eg)/glycerol (Gl)–water	Anti-freezing/self-healing	[95]
Motion detection	UV crosslinking/freeze–thaw cycles	Zwitterionic [2-(methacryloyloxy) ethyl]dimethyl-(3-sulfopropyl) ammonium hydroxide	Adhesive/stretchable/antibacterial	[96]
Motion detection	Oxidative autopolymerization/metal bond coordination	Fe^3+^/catechol-modified chitosan	Adjustable adhesion/toughness/self-healing	[97]
Motion detection	“One-pot” crosslinking procedure	Glycerol–water mixed solvent containing potassium chloride	Adhesiveness/anti-freezing/moisture retention	[98]
Medical diagnostics	Boric acid ester bond	PVA/borax, silk fibroin/TA	Self-healing/self-adhesive	[99]
Medical diagnostics	In situ polymerization	Polyacrylamide	Adhesive/tough	[100]
Medical diagnostics	In situ UV polymerization	Carboxymethyl cellulose/poly acrylic-acrylamide	Elasticity/flexibility	[80]
Medical diagnostics	In situ polymerization	Cellulose	Self-healing/strain/thermal sensitive	[101]
Medical diagnostics	Boric acid ester bond	AgNPs/MXene	Self-healing/injectable/antibacterial	[102]
Electronic skin	Polymerization	Acrylamide	Stretchable/transparent	[103]
Electronic skin	Polymerization	Polymerization of N-isopropylacrylamide/a dopamine-modified polypeptide	Biocompatibility/stable drug release behavior	[104]
Human–machine interactions	Polymerization	ethylene glycol (EG)–water	Anti-freezing	[105]
Human–machine interactions	Polymerization	Poly(sodium acrylate)/MXene	Self-adaptive	[106]

## Data Availability

Not applicable.
